# Supplementary material for: Content-rich biological network constructed by mining PubMed abstracts
Source: BMC Bioinformatics. 2004 Oct 8;5:147. doi: 10.1186/1471-2105-5-147 (PMC528731; doi:10.1186/1471-2105-5-147)
Supplement: Additional File 5 — The original Chilibot query results of the term "long-term potentiation (LTP)" and 22 other terms, limiting the latest references analyzed to the years 1990, 1995, 2000, and 2004. [file 1471-2105-5-147-S5.bz2 › chilibotAdditionalFile5/ltp2004/html/NMDA.html]

 


**NMDA** (Input: NMDA ) 

---


**Google Searches:** Entire Web  | EDU domain only  | PDF files only

.

**External Links:** OMIM |
LocusLink
| Swissprot
| GeneCards

  
**Maps of NMDA**

Simple
Complete
graph in
radiant
tree
square
layout.

**New Hypothesis !**

**Synonyms** 

- nmda   [PubMed]

**Synopsis**

- The results suggest that **NMDA** receptors contribute to the transient increase in 26S proteasome activity during induction of LTP in the hippocampal CA1 region.  Sheng Li Xue Bao, 2003 
  [25]

- These results support the suggestion that low levels of zinc present in experimental solutions may affect the apparent ethanol sensitivity of **NMDA** receptors containing the NR2A subunit.  AlcoholAlcohol, 2003 
  [25]

- The data show that the cell line provides a suitable model to analyse the mechanisms involved in **NMDA** receptor gene expression before the appearance of synaptic communication.  J Neurochem, 2001 
  [23]

- By providing extracellular D serine that facilitates activation of **NMDA** receptors, astrocytes thus play a key role in long term synaptic plasticity.  Proc Natl Acad Sci U S A, 2003 
  [21]

- We present data describing the extracellular stimulation requirements for LTP induction and its long lasting maintenance >4 h at the Schaffer collateral CA1 synapse, and show that such changes in synaptic efficiency are **NMDA** receptor dependent.  J Neurosci Methods, 2003 
  [20]

- These observations provide new information on the stability of developing presynaptic function and suggest that **NMDA** receptor activation may regulate the stability of developing synapses.  J Neurosci, 2002 
  [19]

- Furthermore, the **NMDA** receptor antagonist amino phosphonopentanoic acid APV completely blocked LTD, which suggests a postsynaptic locus of this alteration.  Hippocampus, 2003 
  [19]

- Inhibition of transmitter release in PC 12 cells and **NMDA** treated synaptosomes by immunosuppressants may reflect augmented phosphorylation of NO synthase, reducing its catalytic activity.  Mol Med, 1996 
  [19]

- Because we have previously shown that acute ethanol can inhibit **NMDA** and AMPA,   Alcohol Clin Exp Res, 1999 
  [19]

- The phenotypes of **NMDA** receptor subunits and GluRdelta2 mutant mice suggest that diverse GluR subunits play differential roles in the brain functions.  Life Sci, 2003 
  [19]

- Tests with recombinant **NMDA** receptors NR1 NR2A showed that CX516 and CX546 have no direct effects on currents mediated by these receptors.  Neuroscience, 2004 
  [18]

- Using dendritic and somatic whole cell recordings, combined with calcium imaging in rat hippocampal slices, we found that the induction of long term potentiation  [LTP]  LTP was accompanied by a local increase in dendritic excitability that was dependent on the activation of **NMDA** receptors.  Nat Neurosci, 2004 
  [15]

- Co application of an inositol, 5 trisphosphate IP3 receptor inhibitor, 2 aminotheoxydiphenyl borate, during ACPD application had no effect on the ACPD **NMDA** induced LTP, but increased the magnitude of the ACPD induced LTD, suggesting that the ACPD **NMDA** induced LTP involves **NMDA** receptors,  Brain Res, 2004 
  [15]

- These data indicate that N methyl D aspartate **NMDA** receptor mediated processes are intact at the MPP CA3 synapse in aged rats.  Synapse, 2004 
  [14]

- In addition, the induction of LTP was NOT affected by the N methyl D aspartate **NMDA** receptor antagonist 2 amino 5 phosphonovalerate, suggesting that activation of **NMDA** receptors is NOT required.  Biol Pharm Bull, 2003 
  [14]
